# Age differences in diffusion model parameters: a meta-analysis

**DOI:** 10.1007/s00426-020-01371-8

**Published:** 2020-06-13

**Authors:** Maximilian Theisen, Veronika Lerche, Mischa von Krause, Andreas Voss

**Affiliations:** Psychologisches Institut, Ruprecht-Karls-Universität Heidelberg, Hauptstrasse 47-51, 69117 Heidelberg, Germany

## Abstract

Older adults typically show slower response times in basic cognitive tasks than younger adults. A diffusion model analysis allows the clarification of why older adults react more slowly by estimating parameters that map distinct cognitive components of decision making. The main components of the diffusion model are the speed of information uptake (drift rate), the degree of conservatism regarding the decision criterion (boundary separation), and the time taken up by non-decisional processes (i.e., encoding and motoric response execution; non-decision time). While the literature shows consistent results regarding higher boundary separation and longer non-decision time for older adults, results are more complex when it comes to age differences in drift rates. We conducted a multi-level meta-analysis to identify possible sources of this variance. As possible moderators, we included task difficulty and task type. We found that age differences in drift rate are moderated both by task type and task difficulty. Older adults were inferior in drift rate in perceptual and memory tasks, but information accumulation was even increased in lexical decision tasks for the older participants. Additionally, in perceptual and lexical decision tasks, older individuals benefitted from high task difficulty. In the memory tasks, task difficulty did not moderate the negative impact of age on drift. The finding of higher boundary separation and longer non-decision time in older than younger adults generalized over task type and task difficulty. The results of our meta-analysis are consistent with recent findings of a more pronounced age-related decline in memory than in vocabulary performance.

**Electronic supplementary material:**

The online version of this article (10.1007/s00426-020-01371-8) contains supplementary material, which is available to authorized users.

## Introduction

It is a common finding from the literature on cognitive aging that older people show larger response times (RTs) in basic cognitive tasks than younger adults (Jensen, 2006). In the last decades, the mechanisms underlying this age-related slowing have become a subject of debate. On the one hand, the higher RTs of the older adults might be the result of a general decline in cognitive processing speed due to increased neural noise (Salthouse, 1996). On the other hand, however, it is also possible that the slow responses are based on encoding problems (e.g., due to impaired vision), reduced motoric speed, or more cautious response criteria. Such different accounts can be differentiated by means of diffusion model analyses (Voss, Nagler, & Lerche, 2013). The diffusion model (Ratcliff, 1978) is a stochastic model used to analyze response time distributions and error rates in binary decision tasks. It thus utilizes a more complete representation of decision outcomes than just mean RTs. The model aims to disentangle three main components of the decision process: the speed of information uptake (*drift rate*), the degree of conservatism regarding the decision criterion (i.e., speed-accuracy trade-offs; *boundary separation*) and the time taken up by non-decisional processes such as encoding and motoric response execution (*non-decision time*).

Several diffusion model studies have challenged the view that age differences in RT are indicative of a general decline in cognitive speed (e.g., Spaniol, Madden, & Voss, 2006). Quite often, age differences in RT were not due to differences in the mean speed of information uptake, but due to the fact that older people tended to be more cautious (i.e., they favored accurate over fast responses) and that they took longer in terms of the non-decisional components of the response time (e.g., Ratcliff, Thapar, & McKoon, 2001). However, in some studies, older people additionally showed a lower speed of information uptake (Voskuilen, Ratcliff, & McKoon, 2018), consistent with the notion that processing speed declines with age.

So far, to our knowledge no attempt has been made to bring together the inconsistent results regarding drift rates in a quantitative way. It is an open question whether the discrepancies are simply due to random sample differences or can be explained by specific study attributes. As Dully, McGovern, and O'Connell (2018) note in their literature review, there are “task-specific differences in evidence accumulation rates” (p. 3). However, these task-specific differences have not yet been examined quantitatively.

In this paper, we present the results of a meta-analysis regarding age differences in diffusion model parameters. The focus is on drift rates because of the variability in findings for this parameter. We were interested in whether characteristics of the task (specifically, content and difficulty of task) might explain the inconsistent findings in the literature. We also analyzed the parameters boundary separation and non-decision time. In terms of these parameters, we expected that age differences generalize across tasks. In the next chapter, we briefly introduce the diffusion model (for further introductory information, see e.g., Ratcliff & McKoon, 2008; Voss et al., 2013; Wagenmakers, 2009).

## Introduction to diffusion modeling

The diffusion model is a mathematical model that can be applied to examine the processes underlying RT tasks with two response options. It has most frequently been used with three main task types (Voss et al., 2013). The first group of tasks comprise *memory tasks*. Here, participants usually have to decide whether a stimulus has been presented to them before or not (recognition memory tasks, e.g., Spaniol et al., 2006; Yap, Sibley, Balota, Ratcliff, & Rueckl, 2015). Second, there are *perceptual tasks* in which participants have to discriminate, for example, between two levels of brightness (bright vs. dark, e.g., Ratcliff, 2002; Ratcliff, Thapar, & McKoon, 2003), between two different letters (e.g., F vs. Q, Thapar, Ratcliff, & McKoon, 2003) or between two different quantities of stimuli (small vs. large, e.g., Ratcliff, Thompson, & McKoon, 2015; Ratcliff & Van Dongen, 2009). The third category of task types includes *lexical decision tasks*. In these tasks, participants have to assess whether a presented letter string is a word or not (e.g., Ratcliff, Gomez, & McKoon, 2004; Wagenmakers, Ratcliff, Gomez, & McKoon, 2008).

For these three categories of tasks, it is assumed that the four central assumptions of the diffusion model are met: (1) Information about the two response options is accumulated continuously, (2) decisions are based on single-stage processing, (3) parameters are constant over time, and (4) the tasks are fast response time tasks with mean RTs below 1.5 s. Note, however, that this latter criterion has recently been questioned. Studies demonstrated that also for RT tasks that take up to several seconds per trial, the diffusion model fits well and provides valid parameter estimates (Aschenbrenner, Balota, Gordon, Ratcliff, & Morris, 2016; Lerche, Christmann, & Voss, 2018; Lerche & Voss, 2017). In fact, for such slower tasks, the standard diffusion model that is based on random Gaussian noise might even fit better than for very fast response time tasks (Voss, Lerche, Mertens, & Voss, 2019).

**Fig. 1:**
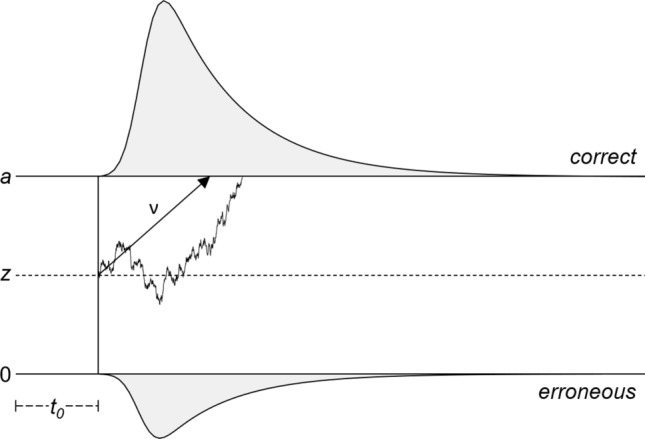
Diffusion model with its four main parameters. The boundaries are associated with correct and erroneous responses here. One exemplary trial is illustrated. In this trial, the accumulation process starts at starting point *z*, which is here right in the center between the two boundaries (0 and *a*). The process moves with speed *ν* toward the upper boundary. To this straight process adds random Gaussian noise. For convenience, parameter *t*_0_ is illustrated at the left from the decision process. Note that it also includes processes succeeding the decisional process (the motoric response)

Four main parameters affect the position and shape of response time distributions in the diffusion model framework. These parameters are also visualized in Fig. 1. First, there is the distance between the two boundaries that are associated with correct (upper boundary) and error responses (lower boundary) in the example figure. This *boundary separation* (*a*) defines the quantity of information that needs to be accumulated before a decision is made. Under accuracy (speed) instructions, participants typically adopt more distant (more close) boundaries (e.g., Ratcliff & Rouder, 1998; Voss, Rothermund, & Voss, 2004). Second, there is the speed of information accumulation, the so-called *drift rate* (*ν*). Drift rate is higher in easier compared to more difficult tasks (e.g., Arnold, Bröder, & Bayen, 2015; Lerche & Voss, 2017) and drift is also positively related to cognitive abilities (e.g., Schmiedek, Oberauer, Wilhelm, Süß, & Wittmann, 2007; Schubert, Hagemann, Voss, Schankin, & Bergmann, 2015).

Third, the *starting point* (*z*) of the accumulation process is modelled. In many tasks, decision processes start from the center between the two boundaries. However, if one of the two response options has a higher expected value (e.g., the response is correct in more trials or higher reward is associated with this response), participants shift the starting point towards the favored option (e.g., Leite & Ratcliff, 2011; Voss et al., 2004).

Finally, *non-decision time* (*t*_0_) subsumes the total duration of all the non-decisional processes (e.g., encoding of information and motor response). Moreover, inter-trial variabilities are often included in the model. However, these variability parameters (in particular, inter-trial variability of drift rate and starting point) often cannot be estimated very reliably (Boehm et al., 2018; Lerche & Voss, 2016; van Ravenzwaaij, Donkin, & Vandekerckhove, 2017) and thus are not very useful to assess inter-individual differences in decision making.

## Methods

As the main focus of this meta-analysis is on examining inconsistent findings concerning age differences in drift rate, the literature search concentrated on studies comparing mean drift rates between two age groups. For these studies, we additionally coded effect sizes for boundary separation and non-decision time. Below, we report our procedure in detail.

### Inclusion criteria and literature search

For our literature search, we used the following two inclusion criteria:

1. All studies had to refer to the original publication introducing the diffusion model in psychology (Ratcliff, 1978) and they had to report results from a diffusion model analysis. Articles applying the EZ-diffusion model (Wagenmakers, van der Maas, & Grasman, 2007) were included. However, we excluded all studies in which parameter estimation was based on fitting the Ex-Gaussian or the shifted Wald distributions due to concerns about the interpretability of their parameters (Matzke & Wagenmakers, 2009).
2. The second required inclusion criterion regards an obligatory comparison between younger adults (college age) and healthy older adults (youngest participant older than 55 or mean age > 60). We excluded studies reporting continuous age analyses if no categorical age data could be extracted from the reported results (e.g., the relation between age and the corresponding parameter of the diffusion model was only provided as a correlation, without raw data being available). We included studies reporting results from more than two age groups if college-aged adults and older adults were among these groups. In case of two higher age groups, we used only the younger one of them to enhance comparability between studies.

We used Google Scholar’s search engine to collect studies, as it allows to combine a descendant approach with the use of specific keywords(for the comparability of Google Scholar with established scientific databases, see Anders & Evans, 2010; Gehanno, Rollin, & Darmoni, 2013; Shultz, 2007). In a first step, we identified all the papers citing Ratcliff’s seminal work on the diffusion model (1978) (*k* = 3341). The next step consisted in searching these studies using age-related terms,^1^ resulting in *k* = 561 publications. The search was finished on January 16, 2019.

We conducted a full-text scan of these papers searching for studies that fulfilled the inclusion criteria, resulting in *k* = 48 articles. After removal of duplicates, *k* = 46 articles remained. Several articles did not report sufficient data to calculate effect sizes on drift rate, resulting in a final dataset of 21 papers. Some papers reported data from more than one sample (e.g., if more than one experiment conducted on different participants is reported in the same publication) and/or more than one effect size per sample (e.g., if different tasks were reported for the same participants). We retrieved effect sizes from 25 samples. For boundary separation and non-decision time, we had to exclude one sample, respectively, as the reported data was not sufficient. In total, we retrieved 146 effect sizes for drift rate, 47 effect sizes for boundary separation, and 40 effect sizes for non-decision time.

### Calculation of effect sizes

As effect size measure we used Hedges’ *g* (Hedges, 1981). We computed effect sizes using the *compute.es* package (version 0.2-4; Del Re, 2013) of the *R* open-source software environment (version 3.5.1; R Core Team, 2018). If a paper did not report means or standard deviations, we used inferential statistics to determine effect sizes. Positive effect sizes indicate higher values for higher age.

### Coding of moderator variables

For each study, we coded the *type of task* using the categories described in Voss, Nagler, and Lerche (2013). Following this classification, most binary decision tasks analyzed with the diffusion model are either perceptual, lexical decision or memory tasks. Some experimental tasks did not fit this classification scheme. We omitted the according effect sizes from the analyses (11 effect sizes for drift rate, 2 effect sizes for boundary separation and 4 effect sizes for non-decision time). See Table 1 for the articles included in this final dataset.

**Table 1:** Samples included in the final dataset of the meta-analysis

Articles	*n*	*n* young	*n* old	Age range young	Age range old	Mean age young	Mean age old
Allen, Lien, Ruthruff, and Voss (2014)	21	11	10	18–24	64–80	21.7	71.8
Ball and Aschenbrenner (2018)	125	67	58	18–21	60–90	18.9	75.0
Dirk et al. (2017)	40	20	20	18–36	64–75	25.7	68.1
Huff and Aschenbrenner (2018)	163	85	78			21	74.6
Kapucu (2010)	56	30	26			19.8	71.9
Kordella (2009)							
Experiment 2	41	22	19	18–24	61–74	20.2	68.9
Experiment 3	38	22	16	18–25	60–74	20.1	68.3
Kühn et al. (2011)	39	24	15	20–31	65–80	25.2	70.2
McKoon and Ratcliff (2012)	78	39	39	18–25	60–74	20.6	68.4
McKoon and Ratcliff (2013)	67	30	37		60–74	20.8	69.7
Ratcliff (2008)	38	19	19		60–75	20.8	69.2
Ratcliff, Thapar, Gomez, and McKoon (2004)							
Experiment 1	98	54	44			19.8	68.5
Experiment 2	94	54	40			20.2	67.2
Ratcliff, Thapar, and McKoon (2004)	80	39	41		60–74	19.6	70
Ratcliff, Thapar, and McKoon (2006)	20	10	10		60–74		
Ratcliff, Thapar, and McKoon (2010)	88	45	43	18–25	60–74	20.8	68.6
Ratcliff, Thapar, and McKoon (2011)	91	46	45		60–74	20.4	68.3
Spaniol, Voss, and Grady (2008)							
Experiment 1	43	22	21	19–28	60–75	22.5	67.5
Experiment 2	47	24	23	18–32	61–85	22.3	71.8
Spaniol, Voss, Bowen, and Grady (2011)	53	26	27	18–32	61–85	23.0	71.5
Thapar, Ratcliff, and McKoon (2003)	78	40	38		60–75	19.8	69.1
Voskuilen et al. (2018)	23	11	12		60–80		

A second moderator variable in our analyses was *task difficulty*. We used drift rate as measure of task difficulty as the literature suggests that more difficult tasks go along with lower drift rates (e.g., Arnold, Bröder, & Bayen, 2015; Voss et al., 2004). In several studies, task difficulty varied between conditions. Here we computed a mean drift rate across the different difficulty levels and age groups (weighting by the number of participants per group). Next, we *z*-transformed and inverted the mean drift so that higher values of the variable indicate enhanced task difficulty.

### Statistical analyses

As several effect sizes are based on the same samples, we assumed effect sizes to be dependent. We accounted for this dependent structure by conducting multilevel meta-analyses using the *metafor* package (version 2.0-0; Viechtbauer, 2010) in *R*. We specified the levels as effect size nested in sample with task type as an inner grouping factor. This means that effects stemming from different samples are assumed to be independent, while effects of the same task type within a sample share correlated random effects. The variance structure of the inner factor was assumed to be a heteroscedastic compound symmetric structure.^2^

We used the maximum likelihood estimation procedure included in the function rma.mv() and analyzed the three outcome variables in independent sets of analyses (i.e., drift rate, boundary separation, and non-decision time). In a first step, we ran multilevel meta-analyses without any moderators (Model 1). Then, in a second step, we included task type and task difficulty as moderators (Model 2). As we were also interested in a possible interaction between task type and task difficulty, we further added the interactions in a third step (Model 3).

The validity of meta-analytical models can suffer because of influential outliers. To date, the development of tools for outlier and influence diagnostics for multilevel meta-analyses is still in progress (Viechtbauer & Cheung, 2010). We followed the procedure of Habeck and Schultz (2015), removing any influential outliers, defined by effect sizes with both hat values greater than two times the average hat value and standardized residual values exceeding 3.29.

We tested for publication bias using Egger’s regression test (Egger, Smith, Schneider, & Minder, 1997; Sterne & Egger, 2005) by modifying Model 1 to include the variance of the effect size as moderator (Moreno et al., 2009). If the intercept of this model significantly deviates from zero, the relationship between variance and effect size can be assumed to be asymmetrical, indicating a bias. Because of the low power of this test for publication bias, we set the alpha-level to *α* = 0.10 (Egger et al., 1997).

Furthermore, we assessed heterogeneity among effect sizes using Cochran’s *Q* statistic and the *I*^2^ statistic. Large *Q* values indicate that differences among effect sizes can be attributed to differences among the true effects and do not solely result from sampling errors. If the *Q* test is significant, the integrated effect size is not an estimator of the true effect but rather an estimator of the mean of the distribution of different true effect sizes (Borenstein, Hedges, Higgins, & Rothstein, 2009).

## Results

### Study characteristics

The included studies stem from the period of 2003–2018. In total, we analyzed the data of 1503 participants (*M* = 62.63 per sample, SD = 34.90). The mean age of the young groups was 21.15 (SD = 1.75), the mean age of the older groups was 69.77 (SD = 2.17). Table 2 shows the distribution of task types over the three diffusion model parameters (see Table S1 in the Supplementary Material for a detailed description of the respective task and condition for each included effect size).

**Table 2:** Number of available effect sizes for each diffusion model parameter depending on the task type

Parameter	Perceptual tasks	Lexical decision tasks	Memory tasks
Drift rate	30	16	89
Boundary separation	16	6	23
Non-decision time	14	6	16

### Diagnostics

For drift rate, there were two cases with standard residual values greater than 3.29. However, their hat values did not exceed 2 and, therefore, we did not omit them from the analyses. For boundary separation and non-decision time, we found no outliers. The intercepts of Egger’s regression models indicated publication bias for all three diffusion model parameters (drift rate: *β*_0_ = 1.049, *p* < 0.001; non-decision time: *β*_0_ = 0.988, *p* < 0.001; boundary separation: *β*_0_ = – 0.637, *p* = 0.063).

### Meta-analysis

#### Drift rate

The meta-analytical model with task type and task difficulty as moderators (Model 2; AICc = 376.2) had a better fit than the model without moderators (Model 1; AICc = 379.1, *p* = 0.022). Including the interaction between task type and difficulty improved the model fit even further (Model 3; AICc = 374.1, *p* = 0.034). Thus, our final meta-analytical model contained task type, task difficulty, and their interaction as moderator variables (see Figure S1 in the Supplementary Material for a forest plot of the final model). For the final model, the *Q* test was highly significant, *Q*(129) = 1016.331, *p* < 0.001. The estimated standard deviations of true effects per task type were τ = 0.848 (perceptual tasks), *τ* = 1.153 (lexical decision tasks), and *τ* = 0.549 (memory tasks). The *I*^2^ values for the three levels of task type were 91.61% (perceptual tasks), 95.28% (lexical decision tasks), and 82.07% (memory tasks).^3^

**Fig. 2:**
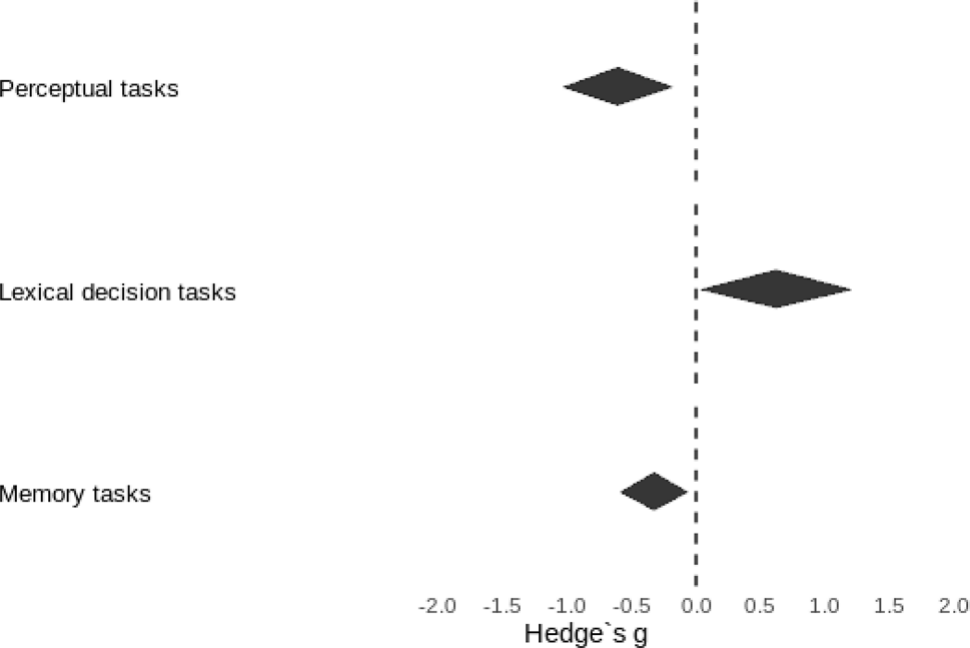
Mean age effects in drift rate for each of the task types analyzed. 95% confidence intervals indicated by the width of the polygons

The mean effect sizes per task type were *g* = − 0.608, 95% CI [− 1.032, − 0.184], *p* = 0.005 for perceptual tasks, *g* = 0.620, 95% CI [0.037, 1.203], *p* = 0.037 for lexical decision tasks and *g* = − 0.326, 95% CI [− 0.587, − 0.065], *p* = 0.014 for memory tasks (see Fig. 2 for a graphical representation). This indicates reduced drift rates for older adults in perceptual and memory tasks but increased drift rates of older adults for lexical decision tasks. Furthermore, in more difficult tasks older adults performed relatively better compared to younger adults (*β* = 0.181, *p* = 0.010).

To examine the task type by task difficulty interaction, we additionally conducted separate analyses for each type of task, with and without task difficulty as moderator. We then compared the fit of the model with and without difficulty to test if this moderator explains variance within a task type (Table 3). For perceptual and lexical decision tasks, the model with difficulty as moderator performed significantly better than the model without moderator. More specifically, task difficulty significantly predicted effect sizes for perceptual (*β* = 0.203, *p* = 0.004) and lexical decision tasks (*β* = 0.719, *p* = 0.022): Older adults profited from high task difficulty. For memory tasks, on the other hand, task difficulty did not predict effect sizes, *β* = 0.016, *p* = 0.816. In the supplementary materials, we provide a full forest plot showing all drift rate effect sizes analyzed.

**Table 3:** Drift rate: comparisons between models with and without task difficulty as moderator for each task type subset

Statistic	Perceptual tasks	Lexical decision tasks	Memory tasks
AICc_with task difficulty_	69.710	63.887	244.189
AICc_without task difficulty_	75.465	65.395	242.100
*p*	.004	.032	.817

#### Boundary separation

The meta-analytical model without any moderators (AICc = 143.8) showed a better fit to the data than the model with task type and task difficulty as moderators (AICc = 149.1, *p* = 0.372). Therefore, we kept the model without any moderators. The mean effect size of age on boundary separation was *g* = 0.731, 95% CI [0.472, 0.989], *p* < 0.001. Results indicate that older adults generally adopt higher boundary separations (i.e., a more conservative response criterion) than young adults. The *Q* test was highly significant, *Q*(44) = 669.203, *p* < 0.001; *I*^*2*^ for the whole model was 93.13%.

#### Non-decision time

The meta-analytical model without any moderators (AICc = 89.9) showed a better fit than the model with task type and task difficulty (AICc = 96.1, *p* = 0.379). Therefore, we kept the model without any moderators. The mean effect size of age on non-decision time was *g* = 1.673, 95% CI [1.404, 1.942], *p* < 0.001. Our results suggest that older people show a longer non-decision time than younger people. The *Q* test was highly significant, *Q*(37) = 388.946, *p* < 0.001; *I*^2^ for the whole model was 90.487%.

## Discussion

In the last decades, the diffusion model (Ratcliff, 1978) has become a popular approach for the analysis of age differences in response time tasks. The findings from the diffusion model analyses have challenged the view that cognitive processing speed generally declines with age. Rather, the studies revealed a more complex picture, which we wanted to examine further in our meta-analysis. Most importantly, we were interested in the drift rate, which is a measure of speed of information accumulation that is closely related to intelligence (e.g., Ratcliff, Thapar, & McKoon, 2011; Schmitz & Wilhelm, 2016). Regarding age effects on drift, previous studies provided inconsistent findings. Whereas some studies report reduced drift rates for older adults (e.g., Thapar et al., 2003), other studies do not find differences in this model parameter (e.g., Ratcliff et al., 2001), or even higher drift rates for older adults (e.g., Ratcliff, Thapar, & McKoon, 2010). With the present meta-analysis, we aim to identify reasons for this heterogeneity. To this aim, we assembled studies that report drift rate differences between a young (college age) and an old age group (> 55 years). Then, we examined the influence of task difficulty and task type (perceptual, lexical decision, and memory) on age effects in diffusion model parameters. Thus, we could uncover possible important moderators that might explain (part of) the inconsistent findings in the literature.

### Boundary separation and non-decision time

Results provided two most clear-cut findings: First, older adults are slower than young adults in non-decisional processes (such as encoding of information and motoric response execution). The corresponding effect size was large (*g* = 1.673). Second, older adults generally use more conservative response criteria (i.e., larger boundary separations) than young adults. Even if the effect size is smaller than for non-decision time, it is still substantial (*g* = 0.731). Thus, older individuals are more cautious in their decisions. These effects did not depend on either task type or task difficulty. Note that both boundary separation and non-decision time influence RT (e.g., Ratcliff & McKoon, 2008). Thus, the common finding of higher RTs of older adults seems to be highly attributable to these two parameters.

### Drift rate

Whereas age differences in boundary separation and non-decision time generalized across different task types and difficulties, we found moderator effects for speed of information accumulation (drift rate). In perceptual tasks and memory tasks, older adults had lower drift rates than younger adults. However, the older groups were superior in speed of information accumulation compared to their younger counterparts in lexical decision tasks. Furthermore, task difficulty also influenced age effects on drift: In terms of perceptual and lexical decision tasks, older participants profited from more difficult tasks. In the memory tasks, task difficulty did not moderate the effect of age on drift.

Thus, our study shows that the pattern of results is clearly more complex for drift rate than for boundary separation and non-decision time and that it seems to be important to consider the specific cognitive processes required by different experimental paradigms. In line with this finding are the results from a recent diffusion model study that is based on a set of 18 different RT tasks (Lerche et al., 2020). The study revealed domain-specific drift factors (numeric, verbal, figural) that were further related to the respective components of an intelligence test. Thus, speed of information accumulation seems to be dependent on the task content. Furthermore, also neurophysiological studies found that aging effects depend on the task (Dully et al., 2018).

Our meta-analysis suggests that older adults outperform young adults in lexical decision tasks, whereas they perform worse in memory tasks. This is in line with the findings from studies that date back to the 1920s and 30s (e.g., Conrad, Jones, & Hsiao, 1933; Foster & Taylor, 1920; Willoughby, 1929). The results of these studies suggest that age differences are more pronounced in measures of memory than vocabulary. Also, recent studies generally confirm this observation. For example, Salthouse (2004), aggregating across several studies from 1998 till 2003, reports a substantial, linear age decline in performance in a memory test. On the other hand, performance improved with age in a vocabulary test, at least until about the mid-50s. After that, it remained stable or somewhat declined (confer also Spaniol et al., 2006). Our meta-analysis showed that such task-specificities are captured in the drift rate of the diffusion model. Furthermore, our analysis revealed that not only the type of task, but also task difficulty needs to be considered. Older adults profited from the more difficult tasks.

## Limitations and future research

Even if the overall number of effect sizes for drift rate used for the meta-analysis was substantial (*N* = 135), analyses of the moderator task type were based on smaller case numbers. Here, the distribution was not balanced with clearly fewer lexical decision effect sizes (*n* = 16) than effect sizes from perceptual (*n* = 30) or memory tasks (*n* = 89). In future research, one might try to replicate our findings in large-scale studies that are explicitly designed to measure the influence of task type (and difficulty) on age differences. Further note that despite consideration of two moderator variables, there was still substantial unexplained variance in our meta-analysis. Accordingly, in future studies, one might try to identify further possible moderators.

The focus of our meta-analysis was on drift rate because findings in the literature seemed to be more inconsistent for this parameter. Therefore, our search strategy was based on finding all studies that report age differences in drift rate. With this strategy we do not identify studies that report age differences only in boundary separation or non-decision time, but not in drift rate. Accordingly, the superiority of the model without moderators might also be partly attributable to the small cell numbers (between 6 and 23 for the different task types). Thus, if one would like to examine moderator influences for non-decision time and boundary separation in more detail, separate meta-analyses should be conducted.

Finally, it would be highly interesting to examine age effects more systematically also for other sequential sampling models, e.g., the popular linear ballistic accumulator model (LBA; Brown & Heathcote, 2008). So far, the literature on age effects in LBA model parameters is more limited than the respective diffusion model literature. The previous LBA findings seem to be generally in line with the results from our meta-analysis: In comparison with younger adults, older adults have been found to have higher threshold separations (Forstmann et al., 2011; Garton, Reynolds, Hinder, & Heathcote, 2019), and longer non-decision times (Ben-David, Eidels, & Donkin, 2014; Garton et al., 2019), whereas the results for drift rate are less clear-cut. Further, previous research suggests that the diffusion model parameters and the LBA model parameters have very similar meanings (Donkin, Brown, Heathcote, & Wagenmakers, 2011). However, to note, in a recent multi-lab project one systematic difference between the two models emerged (Dutilh et al., 2019). More specifically, for instructions that focused either on accuracy or speed the teams that used the diffusion model often found an effect in non-decision time (in addition to an effect in threshold separation), whereas the LBA teams often detected an effect in drift rate. The reasons for this pattern of results will need to be investigated further in future research (for a recent discussion of this topic, see Evans, 2020; Lerche & Voss, 2018). Based on these varying findings, we hypothesize that somehow different age effects might emerge if older and younger adults are compared based on different sequential sampling models. For example, effect sizes for age effects in non-decision time might be larger for the diffusion model than for the LBA.

## Electronic supplementary material

Below is the link to the electronic supplementary material.

Supplementary file1 (PDF 312 kb)

Supplementary file2 (PDF 173 kb)

## Footnotes

1 search string: ("age differences" OR "old adults" OR "old participants" OR "older adult" OR "older adults" OR "older participants" OR "higher age" OR "older group" OR "old group" OR "age-related" OR "effects of aging" OR "effects of age" OR "aging effects" OR "age effects").

2 As recommended by Wolfgang Viechtbauer (Personal communication, April 2018).

3 To compute the *I*^*2*^ on task type level we used the approach for multivariate models as described in Viechtbauer (2018, December 8). To compute the *I*^*2*^ for boundary separation and non-decision time, we used Higgins and Thompson’s (2002) formula.
