# Correction to: Age differences in diffusion model parameters: a meta-analysis

**DOI:** 10.1007/s00426-021-01552-z

**Published:** 2021-07-05

**Authors:** Maximilian Theisen, Veronika Lerche, Mischa von Krause, Andreas Voss

**Affiliations:** Psychologisches Institut, Ruprecht-Karls-Universität Heidelberg, Hauptstrasse 47-51, 69117 Heidelberg, Germany

## Correction to: Psychological Research 10.1007/s00426-020-01371-8

The article “Age differences in diffusion model parameters: a meta-analysis”, written by Maximilian Theisen, Veronika Lerche, Mischa von Krause and Andreas Voss, was originally published electronically on the publisher’s internet portal on 13 June 2020 without open access. With the author(s)’ decision to opt for Open Choice the copyright of the article changed on 7 June 2021 to © The Author(s) 2021 and the article is forthwith distributed under a Creative Commons Attribution 4.0 International License, which permits use, sharing, adaptation, distribution and reproduction in any medium or format, as long as you give appropriate credit to the original author(s) and the source, provide a link to the Creative Commons licence, and indicate if changes were made. The images or other third party material in this article are included in the article’s Creative Commons licence, unless indicated otherwise in a credit line to the material. If material is not included in the article’s Creative Commons licence and your intended use is not permitted by statutory regulation or exceeds the permitted use, you will need to obtain permission directly from the copyright holder. To view a copy of this licence, visit http://creativecommons.org/licenses/by/4.0.

Open access funding enabled and organized by Projekt DEAL.

Original article has been updated.

