# Supplementary material for: Age differences in diffusion model parameters: a meta-analysis
Source: Psychol Res. 2020 Jun 13;85(5):2012–21. doi: 10.1007/s00426-020-01371-8 (PMC8289776; doi:10.1007/s00426-020-01371-8)
Supplement: Supplementary file 1 — Supplementary file1 (PDF 312 kb) [file 426_2020_1371_MOESM1_ESM.pdf]

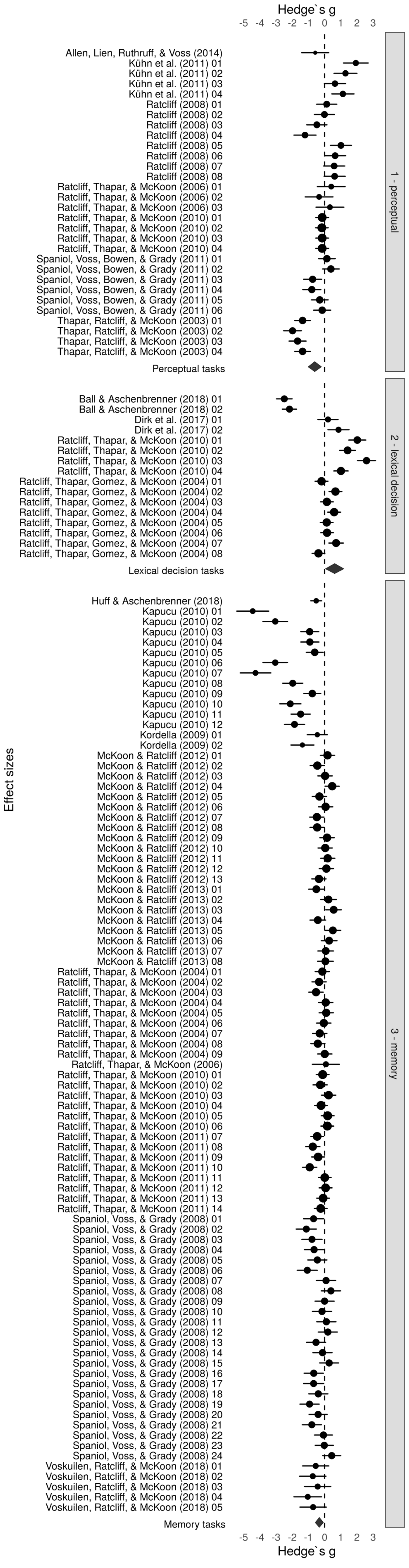

Figure S1. Forest plot of all effect sizes used in the meta-analysis by task type. Points indicate the respective effect size. Point size indicates the proportional weight of the effect size in the meta-analysis. Error bars indicate 95% confidence interval. Diamond center indicates the integrated effect size for each task type. Width of the diamond indicates 95% CI of the integrated effect size. An overview of the specific tasks and conditions associated with the described effect sizes can be found in Table S1. A large amount of variance can be seen in the effect sizes derived from Kapucu (2010, Experiment 3). The task described by Kapucu is a simple memory task where participants had to recognize either positive, neutral, or negative words they had or had not learnt before. The two largest effect sizes were found for negative words that were presented after a delay of 20 minutes. Kapucu does not report any source of systematic variance of age-related differences. A repetition of the meta-analysis without Kapucu’s results lead to the same patterns that were reported for the full dataset.
