# Supplementary material for: Age differences in diffusion model parameters: a meta-analysis
Source: Psychol Res. 2020 Jun 13;85(5):2012–21. doi: 10.1007/s00426-020-01371-8 (PMC8289776; doi:10.1007/s00426-020-01371-8)
Supplement: Supplementary file 2 — Supplementary file2 (PDF 173 kb) [file 426_2020_1371_MOESM2_ESM.pdf]

Table S1

Description of task and condition per effect size included in the final analysis

Task type	Label	Task description	Experimental condition
Perceptual	Allen, Lien, Ruthruff, & Voss (2014)	Green and red discrimination in go/no-go task	
	Kühn et al. (2011) 1	Discrimination of vowels vs. consonants	Before daily cognitive training (100 days)
	Kühn et al. (2011) 2	Discrimination of vowels vs. consonants	After 100 days of training
	Kühn et al. (2011) 3	Discrimination of odd vs. even numbers	Before daily cognitive training (100 days)
	Kühn et al. (2011) 4	Discrimination of odd vs. even numbers	After 100 days of training
	Ratcliff (2008) 1	Numerosity discrimination	31-35 dots
	Ratcliff (2008) 2	Numerosity discrimination	36-40 dots
	Ratcliff (2008) 3	Numerosity discrimination	41-45 dots
	Ratcliff (2008) 4	Numerosity discrimination	46-50 dots
	Ratcliff (2008) 5	Numerosity discrimination	51-55 dots
	Ratcliff (2008) 6	Numerosity discrimination	56-60 dots
	Ratcliff (2008) 7	Numerosity discrimination	61-65 dots
	Ratcliff (2008) 8	Numerosity discrimination	66-70 dots
	Ratcliff, Thapar, & McKoon (2006) 1	Brightness discrimination	
	Ratcliff, Thapar, & McKoon (2006) 2	Letter discrimination	
	Ratcliff, Thapar, & McKoon (2006) 3	Numerosity discrimination	
	Ratcliff, Thapar, & McKoon (2010) 1	Numerosity discrimination	31-35 & 66-70 asterisks
	Ratcliff, Thapar, & McKoon (2010) 2	Numerosity discrimination	36-40 & 61-65 asterisks
	Ratcliff, Thapar, & McKoon (2010) 3	Numerosity discrimination	41-45 & 56-60 asterisks
	Ratcliff, Thapar, & McKoon (2010) 4	Numerosity discrimination	46-50 & 51-55 asterisks
	Spaniol, Voss, Bowen, & Grady (2011) 1	Color discrimination	Control stimulus; gain association
	Spaniol, Voss, Bowen, & Grady (2011) 2	Color discrimination	Control stimulus; loss association
	Spaniol, Voss, Bowen, & Grady (2011) 3	Color discrimination	Neutral stimulus; gain association
	Spaniol, Voss, Bowen, & Grady (2011) 4	Color discrimination	Neutral stimulus; loss association
	Spaniol, Voss, Bowen, & Grady (2011) 5	Color discrimination	Valent stimulus; gain association

Task type	Label	Task description	Experimental condition
Perceptual	Spaniol, Voss, Bowen, & Grady (2011) 6	Color discrimination	Valent stimulus; loss association
	Thapar, Ratcliff, & McKoon (2003) 1	Letter discrimination	Stimulus duration: 10ms
	Thapar, Ratcliff, & McKoon (2003) 2	Letter discrimination	Stimulus duration: 20ms
	Thapar, Ratcliff, & McKoon (2003) 3	Letter discrimination	Stimulus duration: 30ms
	Thapar, Ratcliff, & McKoon (2003) 4	Letter discrimination	Stimulus duration: 40ms
Lexical decision	Ball & Aschenbrenner (2018) 1	Lexical decision task with monitoring of prospective memory cues	Focus on ongoing task
	Ball & Aschenbrenner (2018) 2	Lexical decision task with monitoring of prospective memory cues	Focus on prospective memory cues
	Dirk et al. (2017) 1	Lexical decision task	Non-words
	Dirk et al. (2017) 2	Lexical decision task	Words
	Ratcliff, Thapar, & McKoon (2010) 1	Lexical decision task	High frequency words
	Ratcliff, Thapar, & McKoon (2010) 2	Lexical decision task	Low frequency words
	Ratcliff, Thapar, & McKoon (2010) 3	Lexical decision task	Non-words
	Ratcliff, Thapar, & McKoon (2010) 4	Lexical decision task	Very low frequency words
	Ratcliff, Thapar, Gomez, & McKoon (2004) 1	Lexical decision task	Exp 1: High frequency words
	Ratcliff, Thapar, Gomez, & McKoon (2004) 2	Lexical decision task	Exp 1: Low frequency words
	Ratcliff, Thapar, Gomez, & McKoon (2004) 3	Lexical decision task	Exp 1: Non-words
	Ratcliff, Thapar, Gomez, & McKoon (2004) 4	Lexical decision task	Exp 1: Very low frequency words
	Ratcliff, Thapar, Gomez, & McKoon (2004) 5	Lexical decision task	Exp 2: High frequency words
	Ratcliff, Thapar, Gomez, & McKoon (2004) 6	Lexical decision task	Exp 2: Low frequency words
	Ratcliff, Thapar, Gomez, & McKoon (2004) 7	Lexical decision task	Exp 2: Non-words
	Ratcliff, Thapar, Gomez, & McKoon (2004) 8	Lexical decision task	Exp 2: Very low frequency words

Task type	Label	Task description	Experimental condition
Memory	Huff & Aschenbrenner (2018)	Word recognition	
	Kapucu (2010) 1	Word recognition with and without delay of 20 minutes	Lure words, delay, negative valence
	Kapucu (2010) 2	Word recognition with and without delay of 20 minutes	Lure words, delay, neutral valence
	Kapucu (2010) 3	Word recognition with and without delay of 20 minutes	Lure words, delay, positive valence
	Kapucu (2010) 4	Word recognition with and without delay of 20 minutes	Lure words, no delay, negative valence
	Kapucu (2010) 5	Word recognition with and without delay of 20 minutes	Lure words, no delay, neutral valence
	Kapucu (2010) 6	Word recognition with and without delay of 20 minutes	Lure words, no delay, positive valence
	Kapucu (2010) 7	Word recognition with and without delay of 20 minutes	Target words, delay, negative valence
	Kapucu (2010) 8	Word recognition with and without delay of 20 minutes	Target words, delay, neutral valence
	Kapucu (2010) 9	Word recognition with and without delay of 20 minutes	Target words, delay, positive valence
	Kapucu (2010) 10	Word recognition with and without delay of 20 minutes	Target words, no delay, negative valence
	Kapucu (2010) 11	Word recognition with and without delay of 20 minutes	Target words, no delay, neutral valence
	Kapucu (2010) 12	Word recognition with and without delay of 20 minutes	Target words, no delay, positive valence
	Kordella (2009) 1	Sentence memory	Exp 2: Sentence recognition
	Kordella (2009) 2	Sentence memory	Exp 3: Source memory
	McKoon & Ratcliff (2012) 1	Associative word recognition	Intact from related word pairs
	McKoon & Ratcliff (2012) 2	Associative word recognition	Intact from non-related word pairs
	McKoon & Ratcliff (2012) 3	Associative word recognition	Rearranged from non-related word pairs
	McKoon & Ratcliff (2012) 4	Associative word recognition	Rearranged from related word pairs
	McKoon & Ratcliff (2012) 5	Word recognition	High frequency words, new
	McKoon & Ratcliff (2012) 6	Word recognition	High frequency words, old

Task type	Label	Task description	Experimental condition
Memory	McKoon & Ratcliff (2012) 7	Word recognition	Low frequency words, new
	McKoon & Ratcliff (2012) 8	Word recognition	Low frequency words, old
	McKoon & Ratcliff (2012) 9	Word recognition	Primed from non-related word pairs
	McKoon & Ratcliff (2012) 10	Word recognition	Primed from related pairs
	McKoon & Ratcliff (2012) 11	Word recognition	Primes
	McKoon & Ratcliff (2012) 12	Word recognition	Unprimed from non-related word pairs
	McKoon & Ratcliff (2012) 13	Word recognition	Unprimed from related word pairs
	McKoon & Ratcliff (2013) 1	Word recognition after reading sentences with related inferences	Control
	McKoon & Ratcliff (2013) 2	Word recognition after reading sentences with related inferences	Filler from control sentence
	McKoon & Ratcliff (2013) 3	Word recognition after reading sentences with related inferences	Filler from predicting sentence
	McKoon & Ratcliff (2013) 4	Word recognition after reading sentences with related inferences	New word
	McKoon & Ratcliff (2013) 5	Word recognition after reading sentences with related inferences	Predicting
	McKoon & Ratcliff (2013) 6	Word recognition after reading sentences with related inferences	Prime for control sentence
	McKoon & Ratcliff (2013) 7	Word recognition after reading sentences with related inferences	Prime for predicting sentence
	McKoon & Ratcliff (2013) 8	Word recognition after reading sentences with related inferences	Word from filler sentence
	Ratcliff, Thapar, & McKoon (2004) 1	Word recognition	High frequency words presented once
	Ratcliff, Thapar, & McKoon (2004) 2	Word recognition	High frequency words presented three times
	Ratcliff, Thapar, & McKoon (2004) 3	Word recognition	Low frequency words presented once
	Ratcliff, Thapar, & McKoon (2004) 4	Word recognition	Low frequency words presented three times
	Ratcliff, Thapar, & McKoon (2004) 5	Word recognition	New high frequency words
	Ratcliff, Thapar, & McKoon (2004) 6	Word recognition	New low frequency words
	Ratcliff, Thapar, & McKoon (2004) 7	Word recognition	New very low frequency words
	Ratcliff, Thapar, & McKoon (2004) 8	Word recognition	Very low frequency words presented once

Task type	Label	Task description	Experimental condition
Memory	Ratcliff, Thapar, & McKoon (2004) 9	Word recognition	Very low frequency words presented three times
	Ratcliff, Thapar, & McKoon (2006)	Word recognition	
	Ratcliff, Thapar, & McKoon (2010) 1	Word recognition	High frequency words presented once
	Ratcliff, Thapar, & McKoon (2010) 2	Word recognition	High frequency words presented twice
	Ratcliff, Thapar, & McKoon (2010) 3	Word recognition	Low frequency words presented once
	Ratcliff, Thapar, & McKoon (2010) 4	Word recognition	Low frequency words presented twice
	Ratcliff, Thapar, & McKoon (2010) 5	Word recognition	New high frequency words
	Ratcliff, Thapar, & McKoon (2010) 6	Word recognition	New low frequency words
	Ratcliff, Thapar, & McKoon (2011) 7	Associative word recognition	High frequency words from intact pairs
	Ratcliff, Thapar, & McKoon (2011) 8	Associative word recognition	High frequency words from rearranged words
	Ratcliff, Thapar, & McKoon (2011) 9	Associative word recognition	Low frequency words from intact pairs
	Ratcliff, Thapar, & McKoon (2011) 10	Associative word recognition	Low frequency words from rearranged words
	Ratcliff, Thapar, & McKoon (2011) 11	Word recognition	New high frequency words
	Ratcliff, Thapar, & McKoon (2011) 12	Word recognition	New low frequency words
	Ratcliff, Thapar, & McKoon (2011) 13	Word recognition	Old high frequency words
	Ratcliff, Thapar, & McKoon (2011) 14	Word recognition	Old low frequency words
	Spaniol, Voss, & Grady (2008) 1	Item recognition	Exp 1: New faces with negative valence
	Spaniol, Voss, & Grady (2008) 2	Item recognition	Exp 1: New faces with neutral valence
	Spaniol, Voss, & Grady (2008) 3	Item recognition	Exp 1: New faces with positive valence
	Spaniol, Voss, & Grady (2008) 4	Item recognition	Exp 1: New scenes with negative valence
	Spaniol, Voss, & Grady (2008) 5	Item recognition	Exp 1: New scenes with neutral valence
	Spaniol, Voss, & Grady (2008) 6	Item recognition	Exp 1: New scenes with positive valence
	Spaniol, Voss, & Grady (2008) 7	Item recognition	Exp 1: New words with negative valence
	Spaniol, Voss, & Grady (2008) 8	Item recognition	Exp 1: New words with neutral valence
	Spaniol, Voss, & Grady (2008) 9	Item recognition	Exp 1: New words with positive valence
	Spaniol, Voss, & Grady (2008) 10	Item recognition	Exp 1: Old faces with negative valence
	Spaniol, Voss, & Grady (2008) 11	Item recognition	Exp 1: Old faces with neutral valence

Task type	Label	Task description	Experimental condition
Memory	Spaniol, Voss, & Grady (2008) 12	Item recognition	Exp 1: Old faces with positive valence
	Spaniol, Voss, & Grady (2008) 13	Item recognition	Exp 1: Old scenes with negative valence
	Spaniol, Voss, & Grady (2008) 14	Item recognition	Exp 1: Old scenes with neutral valence
	Spaniol, Voss, & Grady (2008) 15	Item recognition	Exp 1: Old scenes with positive valence
	Spaniol, Voss, & Grady (2008) 16	Item recognition	Exp 1: Old words with negative valence
	Spaniol, Voss, & Grady (2008) 17	Item recognition	Exp 1: Old words with neutral valence
	Spaniol, Voss, & Grady (2008) 18	Item recognition	Exp 1: Old words with positive valence
	Spaniol, Voss, & Grady (2008) 19	Item recognition	Exp 2: New scenes with negative valence
	Spaniol, Voss, & Grady (2008) 20	Item recognition	Exp 2: New scenes with neutral valence
	Spaniol, Voss, & Grady (2008) 21	Item recognition	Exp 2: New scenes with positive valence
	Spaniol, Voss, & Grady (2008) 22	Item recognition	Exp 2: Old scenes with negative valence
	Spaniol, Voss, & Grady (2008) 23	Item recognition	Exp 2: Old scenes with neutral valence
	Spaniol, Voss, & Grady (2008) 24	Item recognition	Exp 2: Old scenes with positive valence
	Voskuilen, Ratcliff, & McKoon (2018) 1	Word recognition	High frequency words presented in training for 1.5 s
	Voskuilen, Ratcliff, & McKoon (2018) 2	Word recognition	High frequency words presented in training for 5 s
	Voskuilen, Ratcliff, & McKoon (2018) 3	Word recognition	Low frequency words presented in training for 1.5 s
	Voskuilen, Ratcliff, & McKoon (2018) 4	Word recognition	Low frequency words presented in training for 5 s
	Voskuilen, Ratcliff, & McKoon (2018) 5	Word recognition	New low frequency words
